# Pulsus alternans under left ventricular assist device in a patient with dilated cardiomyopathy and *LMNA* mutation

**DOI:** 10.1093/eurheartj/ehae485

**Published:** 2024-08-05

**Authors:** Ryohei Sakai, Takeshi Kashimura, Takayuki Inomata

**Affiliations:** Department of Cardiovascular Medicine, Niigata University Medical and Dental Hospital, 1-754 Asahimachidori, Chuoku, Niigata 951-8520, Japan; Department of Cardiovascular Medicine, Niigata University Medical and Dental Hospital, 1-754 Asahimachidori, Chuoku, Niigata 951-8520, Japan; Department of Advanced Cardiopulmonary Vascular Therapeutics, Niigata University Graduate School of Medical and Dental Sciences, 1-757 Asahimachidori, Chuo-ku, Niigata 951-8510, Japan; Department of Cardiovascular Medicine, Niigata University Medical and Dental Hospital, 1-754 Asahimachidori, Chuoku, Niigata 951-8520, Japan

A 50-year-old man with dilated cardiomyopathy who had been treated with optimal medical therapy for 5 years and cardiac resynchronizing therapy with a defibrillator for 1 year for bradycardic atrial fibrillation presented with progressive heart failure with a left ventricular ejection fraction of 22% and skeletal muscle weakness. Left ventricular endomyocardial biopsy revealed distorted myocardial nuclei (*Panel A*) and interstitial fibrosis (*Panel B*), and blood sample analysis revealed a truncation mutation of *LMNA* (*Panel C*).

**Figure:**
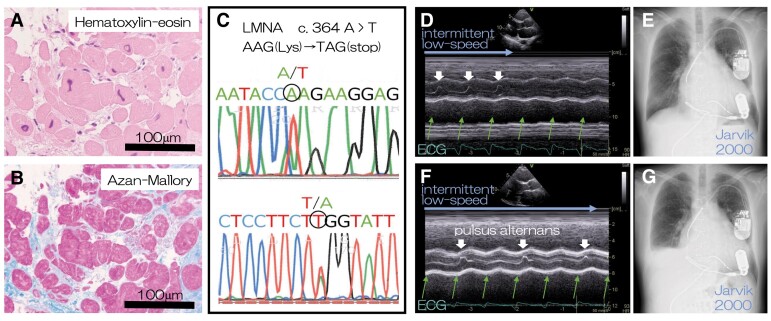


He underwent implantation of a left ventricular assist device (LVAD), Jarvik 2000, with an 8 s intermittent low-speed (ILS) mode in each 64 s period for facilitating aortic valve opening. The aortic valve opened with each heartbeat only during the ILS mode (*Panels D and E*).

However, 2 years later, echocardiography during the ILS mode showed alternating aortic valve opening or pulsus alternans (*Panel F*; Supplementary data online, *Video S1*). Two months later, the patient presented with dyspnoea and oedema; chest radiography revealed pleural effusion (*Panel G*). Right-sided heart failure progressed continuously, and the patient died 1 year later.

The mutation of *LMNA*, which encodes lamins A and C that localize to the inner nuclear membrane, may cause myocardial nuclear deformity and dysfunction.^1^ Pulsus alternans is presumably attributed to myocardial calcium–handling failure, which is one of the proposed mechanisms of myocardial dysfunction with *LMNA* mutation.^2^

Recently, pulsus alternans was reported in right-sided heart failure under peripheral LVAD in a patient with acute myocarditis.^3^ However, whether pulsus alternans under LVAD is a sign of right-sided heart failure or typical in *LMNA* mutation should be elucidated in future studies.

Supplementary data are available at *European Heart Journal* online.

All authors declare no disclosure of interest for this contribution.

No data were generated or analysed for or in support of this paper.

## Supplementary Material

ehae485_Supplementary_Data
